# Changes in prevalence, awareness, treatment and control of hypertension from 2004 to 2014 among 25-74-year-old citizens in the Yangon Region, Myanmar

**DOI:** 10.1186/s12889-017-4870-y

**Published:** 2017-10-26

**Authors:** Aung Soe Htet, Marius B. Bjertness, Win Myint Oo, Marte Karoline Kjøllesdal, Lhamo Y. Sherpa, Ko Ko Zaw, Ko Ko, Hein Stigum, Haakon E. Meyer, Espen Bjertness

**Affiliations:** 1 Department of Community Medicine and Global Health, Institute of Health and Society, Faculty of Medicine, University of Oslo, Oslo, Norway; 2 International Relations Division, Ministry of Health, Nay Pyi Taw, Myanmar; 3 Faculty of Medicine, SEGi University, Petaling Jaya, Malaysia; 4 University of Public Health, Yangon, Myanmar; 5 Department of Diabetes and Endocrinology, University of Medicine 2, Yangon, Myanmar

**Keywords:** Hypertension, Prevalence, Awareness, Treatment, Control, Yangon, Myanmar

## Abstract

**Background:**

Hypertension is the leading risk factor for cardiovascular diseases, and little is known about trends in prevalence, awareness, treatment and the control of hypertension in Myanmar. This study aims at evaluating changes from 2004 to 2014 in the prevalence, awareness, treatment and control of hypertension in the Yangon Region, Myanmar, and to compare associations between hypertension and selected socio-demographic, behavioural- and metabolic risk factors in 2004 and 2014.

**Methods:**

In 2004 and 2014, household-based cross-sectional studies were conducted in urban and rural areas of Yangon Region using the WHO STEPS protocol. Through a multi-stage cluster sampling method, a total of 4448 and 1486 participated in 2004 and 2014, respectively, with the response rates above 89%.

**Results:**

From 2004 to 2014, there was a significant increase in the age-standardized prevalence of hypertension from 26.7% (95% CI:24.4-29.1) – 34.6% (32.2-37.1), as well as an awareness from 19.4% (17.2-21.9) to 27.8% (24.9-31.0), while treatment and control rates did not change. The age-standardized mean systolic blood pressure increased from 122.8 (SE) ± 0.82 mmHg in 2004 to 128.1 ± 0.53 mmHg in 2014, whereas diastolic blood pressure increased from 76.2 ± 0.35 mmHg to 80.9 ± 0.53 mmHg. In multivariate analyses, hypertension was significantly associated with age, alcohol consumption, overweight and diabetes in both 2004 and 2014, and additionally associated with low physical activity and hypercholesterolemia in 2004. Combining all data, a significant association between study-year and hypertension persisted in different models with an adjustment for socio-demographic variables and behavioural variables, but not when adjusting for a combination of socio-demographic variables, the metabolic variables, BMI and hypercholesterolemia.

**Conclusion:**

The prevalence of hypertension has risen from 2004 to 2014 in both urban and rural areas of the Yangon Region, while, the awareness, treatment and control rate of hypertension remains low in urban and rural areas among both males and females. It is likely that changes in the metabolic variables, BMI and hypercholesterolemia have contributed to an increase in the prevalence of hypertension from 2004 to 2014. Factors associated with hypertension in both study years were age, alcohol consumption, overweight and diabetes. A national hypertension control programme should be implemented in order to reduce premature deaths in Myanmar.

**Electronic supplementary material:**

The online version of this article (10.1186/s12889-017-4870-y) contains supplementary material, which is available to authorized users.

## Background

Globally, high systolic blood pressure is the leading risk factor for years lost due to ill-health, disability or early death (DALY), in addition to the most important risk factors for cardiovascular diseases (CVDs) together with dietary risks [1]. High systolic blood pressure has been estimated to cause 10.7 million deaths and 212 million DALYs in 2015 [1], and 58% of CVDs in 2013 globally [2]. Although we have knowledge about how to prevent and treat hypertension, its prevalence has not decreased over the last four decades in large parts of the world, including southeast Asia [3]. Globally, the number of individuals with hypertension has increased from 594 million in 1975 to 1.13 billion in 2015 [3], which is partly due to population growth and the aging of the global populations by more than 10 years since 1980 [4], combined with a failure in the prevention, diagnosis and control of the disorder [5]. In Southeast Asia, 75% or more of the rise in cases with hypertension are attributable to population growth and ageing, while 25% is due to an increase in prevalence [3]. The United Nations declaration of Sustainable Development Goals (SDGs) targets calls for a one-third reduction in premature mortality from non-communicable diseases (NCDs) through prevention and treatment by 2030. In 2013, the 66th World Health Assembly targeted a 25% relative reduction in the prevalence of raised blood pressure by 2025 [6].

The prevalence of hypertension is higher in low-income countries than in middle- and high-income countries [7], as the risk of dying from hypertension is more than double in low- and middle- compared to high income countries [8].

In Southeast Asia, hypertension is estimated to cause 9.4% of all deaths [8]. The age-standardized prevalence of hypertension in the region was estimated to be 36.6% in 2008 [9], with country estimates of 34.2% in Thailand, 38.6% in Bangladesh and 38.8% in Nepal [9]. The corresponding levels of awareness, treatment and the control of hypertension in these countries varied considerably, respectively, at 56.6, 85.5 and 43.1% in Thailand, 69.8, 48.4 and 42.2% in Bangladesh and 29.7, 41.2 and 22.3% in Nepal [9]. In Myanmar, the prevalence of hypertension was reported to be 30% in a nationwide study from 2009 [10]. Studies from SEAR have shown an increasing trend in the prevalence of hypertension [9].

Information about trends in prevalence, awareness, treatment and the control of hypertension, which is largely unknown in Myanmar [10, 11], could help in tailoring prevention and treatment programmes within the country.

In the present study, we analysed changes in the prevalence, awareness, treatment and the control of hypertension from 2004 to 2014 in the Yangon region of Myanmar. Furthermore, we compared associations between hypertension and socio-demographic-, behavioural and metabolic risk factors in 2004 and 2014. Finally, we aimed at investigating associations between the year of study and hypertension. It is expected that the increase in hypertension from 2004 to 2014 could be explained by socio-demographic, behavioural and metabolic risk factors.

## Methods

In 2003-2004 and 2013-2014, two cross-sectional studies were carried out in the Yangon region [11, 12]. Data was collected in both the urban and rural areas in two study periods. The methods of both studies were similar, and conducted in accordance with the WHO STEPS methodology [13]. Both studies included all three STEPS: STEP (1) Questionnaire survey, including the socio-demographic characteristics, behaviours and dietary habits, and history of hypertension and diabetes; STEP (2) Physical measurements, including anthropometric measurements and blood pressure, and STEP (4) A laboratory investigation of fasting blood glucose and fasting lipid profiles.

The study populations included both males and females aged 25-74 years. Individuals who were too physically or mentally ill to participate, institutionalized people, military personnel, Buddhist monks and nuns were excluded.

Both studies performed a multi-stage cluster sampling method. In the first stage, urban and rural areas were identified. In the second stage, 10 townships from the urban areas and five townships from the rural areas were randomly selected in 2004, while six townships from urban- and six townships from rural areas were randomly selected in 2014. In the third stages, 10 wards (urban unit) and 10 villages (rural unit), respectively, from each of the urban and rural townships were randomly selected in 2004. In 2014, five wards and five villages from each of the urban and rural townships were randomly selected. In the fourth stage, both in 2004 and 2014, 25-27 households were randomly chosen from the selected clusters (ward/village). Gender stratification was done in this stage in 2014, but not in 2004. Lastly, one eligible subject was randomly selected from each household.

A total of 4448 persons participated in 2004 and 1486 in 2014. The response rates were 89% in 2004 and 92% in 2014 for STEP 1 and 2, while 89 and 86% complete all three STEPS in 2004 and 2014, respectively. Pregnant women were excluded from the analysis because physiological changes during pregnancy affected the estimates.

### Data collection and measurements

The STEPS instrument (version 1.3) was translated into the Myanmar language and back translated into English in 2004. Previously translated questionnaires (version 2.1) were used in 2014. Compared to version 1.3, version 2.1 included more core and expanded questions. The training of field workers, pre-test and pilots were conducted in a similar way in both 2004 and 2014. We used identical methods for training and for practice in both surveys following the STEPS methodology [13]. The measurement of blood pressure was performed three times using the automatic blood pressure monitor OMRON M4-1 (Japan) in 2004 (in 2004, two times only if the difference was less than 10 mmHg) and validated OMRON M6 (Japan) [14] in 2014. The accuracy for the both devices was ±3 mmHg according to OMRON manual. Systolic and diastolic blood pressures were computed from the mean of all readings in 2004 and second and third readings in 2014. Body height was measured without footwear and any head gear using stadiometer (which was supported by WHO SEARO) in 2004 and measuring tape (SPEED-5 M) in 2014 [13]. In both studies, a portable weighting scale (SECA 843 in 2004 and Equinox BR-9808 in 2014) was used to measure the body weight of participants in kilograms (kg) wearing light clothes without any footwear.

Fasting venous blood samples were collected in a nearby health facility or survey site after a minimum of 10 h of overnight fasting, using lipid tubes and glucose tubes containing fluoride. The samples were stored in cold boxes with ice and transported to the laboratory at the Department of Medical Research, Yangon in 2004 and to the National Health Laboratory, Yangon, which is the reference laboratory in Myanmar, in 2014. A laboratory investigation was conducted within 3 hours of the blood collection.

The blood glucose concentration was determined by an enzymatic colourimetric test, using reagents from Human Gesellschaft fur Biochemica und Diagnostica mbh, Germany in 2004, and by the enzymatic reference method with hexokinase, using reagents from Roche Diagnostics, Indianapolis, USA in 2014. Serum concentrations of total cholesterol and triglycerides were determined using the enzymatic colourimetric test with reagents from Human Gesellschaft fur Biochemica und Diagnostica mbh, Germany in 2004, and with reagents from Roche Diagnostics, Indianapolis USA in 2014.

### Variables

*Hypertension* was defined as a systolic blood pressure of ≥140 mmHg and/or a diastolic blood pressure of ≥90 mmHg, and/or a self-reported current use of anti-hypertensive medication within 2 weeks prior to the interview [13]. An *awareness of hypertension* was defined as the knowledge of being hypertensive obtained through a medical doctor or health worker. The *treatment of hypertension* was defined in those who had taken anti-hypertension drugs for at least the preceding 2 weeks to the time of interview. We defined *control of hypertension* as those under medication who presented a systolic blood pressure of <140 mmHg and diastolic blood pressure of <90 mmHg.

In accordance with the ward or village tract Administration Law of 2012 [15], we define a ward as an urban unit and a village as a rural unit.

*Level of education* was categorized into four groups: no formal school (0- years), primary level (1-5 years), secondary and high school level (6-11 years) and higher education level (12 years and above). Yearly household *income* was calculated from the household income earned by household members in local currency (Kyats).

A *daily smoker* was defined as those who currently smoked tobacco everyday. A *current alcohol drinker* was defined as those who have consumed alcohol within the past 12 months.

A physical activity assessment was conducted from the four domains of occupation, household chores, transportation and leisure time. A *low physical activity* was defined by using the standard metabolic equivalent of tasks (MET) based on the WHO guidelines as less than 3 days of vigorous intensity activity at least 20 min per week, or less than 5 days of moderate intensity activity per week (with ≥600 MET- minutes) [13].

*Body Mass Index* (BMI) was computed as weight in kilograms (kg) divided by the height in metres squared (m^2^). *Overweight* was defined as a BMI of 25-29.9 kg/m^2^, with obesity defined as a BMI of ≥30 kg/m^2^ [13]. We defined *diabetes* as a fasting blood glucose level of ≥7 mmol/L and/or self-reported diabetes [16]. Self-reported diabetes included only those diagnosed by health professionals. We defined hypercholesterolemia as a total cholesterol level of ≥5.17 mmol/L [17].

### Statistical methods

Data from 2004 and 2014 was analysed with STATA/IC version 14.0. We adjusted for the complex survey design using the “svyset” command with a specified weighing of different stages of sampling units based on the population distribution of age and gender in urban and rural study sites of the Yangon region. For the 2004 study, we used the population distribution in 2003 [18], while for the 2014 study we used census data from 2014 [19]. Following the “svy” command, mean and proportions were computed incorporating the complex survey design. Prevalences of hypertension, awareness, treatment and control in 2004 and 2014 were standardized to the population age distribution in 2014 (Census) due to expected differences in age distribution between 2004 and 2014. A comparison of the proportions and means between the two studies was performed by Wald test. Following the “svy” command, multivariate logistic regressions were performed to compare associations between hypertension and socio-demographic-, behaviuoral- and metabolic risk factors in 2004 and 2014 (Tables 4 and 5.). In Model 1, the associations between hypertension and selected behavioural- and metabolic risk factors were adjusted one by one for the potential socio-demographic confounders of age, gender, location, education and ethnicity. Thus, Model 1 gives the total effect of each of the selected behaviuoral and metabolic determinants of hypertension adjusted for socio-demographic factors. In Model 2, both socio-demographic- and behavioural risk factors (smoking, alcohol consumption, low physical activity) were included, and all the variables in the model were adjusted for. In Model 3, the metabolic risk factors of BMI, diabetes and hypercholesterolemia were included together with the socio-demographic factors, and all variables in the model were adjusted for. In Model 4, we adjusted for all variables mentioned above, which gives the direct effect of each of the selected behavioural and metabolic determinants of hypertension. We checked for interactions between gender and smoking on hypertension as well as between location and gender on hypertension. For the purpose of investigating whether the expected association between the year of study and hypertension could be explained by socio-demographic-, behavioural- and metabolic risk factors, we performed logistic regression analyses following the “svy” command (Table 6). In Model 1, we adjusted for age and gender, and in Model 2 we also adjusted for location, education and ethnicity. In Model 3, we additionally adjusted for the behavioural risk factors (smoking, alcohol consumption, low physical activity). In Model 4, we adjusted for metabolic risk factors (BMI, diabetes and hypercholesterolemia), in addition to the socio-demographic risk factors from Model 2. In Model 5, we adjusted for all selected socio-demographic-, behavioural- and metabolic risk factors. We did six additional logistic regression analyses to identify which of the socio-demographic-, behavioral- or metabolic risk factors that could make the association between study year and hypertension become non-significant and/or reduced towards the null value (|Additional file 1: Table S1). We used the following six models (socio-demographic factors were always included): 1. BMI; 2. diabetes; 3. hypercholesterolemia; 4. BMI and diabetes; 5. diabetes and hypercholesterolemia; and 6. BMI and hypercholesterolemia.

## Results

The mean age of the participants was lower in 2014 (47.6 years) than in 2004 (49.9 years), whereas the levels of education and income were highest in 2014 (Table 1).

**Table 1:** Socio-demographic factors among 25-74-year-old citizens in Yangon Region, Myanmar, in 2004 and 2014

		2004	2014	*p*-value
Gender				
Male		1994	745	
Female		2454	741	
Age (Mean Years ± SD)				
Male		49.9 ± 13.3	48.4 ± 13.3	0.001 *
Female		48.7 ± 12.7	46.9 ± 12.4	0.131
Total		49.2 ± 13.0	47.6 ± 12.9	0.001 *
Age group (proportion %)				
25-34		707 (15.9)	286 (19.2)	
35-44		945 (21.3)	350 (23.6)	
45-54		1131 (25.4)	351 (23.6)	
55-64		981 (22.1)	329 (22.1)	
65-74		684 (15.4)	170 (11.4)	0.526
Highest Education Level (proportion %)				
No formal school		354 (7.96)	90 (6.1)	
Less than Primary school		840 (18.9)	223 (15.0)	
Primary school completed		1548 (34.8)	468 (31.5)	
Secondary school completed		929 (20.9)	278 (18.7)	
High school completed		429 (9.6)	186 (12.5)	
College/university completed		320 (7.2)	211 (14.2)	
Post graduate degree		28 (0.6)	28 (1.9)	<0.001 *
Yearly household Income (proportion %) ^$^				
Mean (Kyats)		44,968	259,605	
Less than 100,000 Kyats		3963 (95.1)	481 (34.5)	
100,001- < 150,000 Kyats		81 (1.9)	268 (19.2)	
150,001- < 300,000 Kyats		106 (2.5)	404 (29.0)	
> 300,000 Kyats		21 (0.5)	242 (17.4)	

^$^ *Exchange rate 1 USD = 750 Kyats in 2004 and 953.8 Kyats in November 2013*

The age-standardized prevalence of hypertension increased significantly from 26.7% (95% CI: 24.4-29.1) in 2004 to 34.6% (32.2-37.1) in 2014 (Table 2). Similar significant increases from 2004 to 2014 were found in sub-groups of males and females and in urban and rural locations (Table 2).

**Table 2:** Age-standardized prevalence of hypertension, and awareness, treatment and control of hypertension among 25-74-year-old citizens of Yangon Region, in 2004 and 2014, by gender and location (Standardized to the 2014 Census Data of Yangon Region)

	Male			Female			Total		
	2004 (*N* = 1994)	2014 (*N* = 745)	*p*-value	2004 (*N* = 2454)	2014 (*N* = 741)	*p*-value	2004 (*N* = 4448)	2014 (*N* = 1486)	*p*-value
Prevalence									
Combined	26.8 (24.1-29.8)	36.6 (32.1-41.2)	0.001*	26.6 (24.2-29.1)	33.6 (30.8-36.6)	<0.001*	26.7 (24.4-29.1)	34.6 (32.2-37.1)	<0.001*
Urban	27.9 (23.9-32.4)	38.7 (33.9-43.7)	0.003*	27.7 (24.4-31.3)	34.5 (28.9-40.6)	0.050*	27.6 (24.3-31.1)	34.5 (31.5-37.6)	0.006*
Rural	24.0 (19.5-29.1)	33.4 (27.4-40.1)	0.025*	24.0 (20.8-27.5)	33.8 (30.7-37.1)	0.001*	24.3 (21.4-27.5)	34.2 (30.0-38.6)	0.002*
Awareness									
Combined	15.0 (12.8-17.4)	23.0 (19.9-26.5)	<0.001*	22.8 (19.9-25.9)	32.5 (29.1-36.1)	<0.001*	19.4 (17.2-21.9)	27.8 (24.9-31.0)	<0.001*
Urban	15.2 (12.6-18.3)	22.6 (19.0-26.7)	0.005*	24.3 (20.1-29.0)	31.5 (26.3-37.3)	0.045*	20.6 (17.4-24.3)	26.9 (22.3-31.9)	0.039*
Rural	14.1 (11.1-17.8)	23.5 (19.5-28.1)	0.003*	18.8 (17.8-19.9)	32.7 (33.8-38.7)	<0.001*	16.5 (14.7-18.5)	28.5 (23.8-33.7)	<0.001*
Treatment									
Combined	38.0 (33.8-42.5)	38.6 (31.6-46.1)	0.891	48.1 (39.7-56.6)	47.2 (40.8-53.7)	0.865	43.1 (34.8-51.9)	40.1 (33.1-47.6)	0.589
Urban	41.9 (34.5-49.6)	46.7 (39.6-54.0)	0.339	52.2 (43.2-61.0)	55.6 (49.7-61.2)	0.504	47.5 (37.4-57.8)	46.3 (35.4-57.5)	0.861
Rural	35.5 (27.6-44.2)	41.8 (33.3-50.9)	0.271	38.0 (33.2-42.9)	45.9 (40.2-51.8)	0.042*	36.9 (30.4-43.9)	40.0 (32.4-48.1)	0.522
Control									
Combined	35.8 (31.5-40.3)	39.8 (34.7-45.2)	0.234	53.0 (49.0-57.0)	47.3 (44.7-49.8)	0.019*	48.4 (45.1-51.7)	45.3 (39.8-50.9)	0.330
Urban	37.8 (30.8-45.3)	39.1 (33.5-45.0)	0.763	52.4 (46.7-58.1)	43.4 (40.1-46.7)	0.010*	50.9 (46.3-55.5)	41.6 (38.4-44.8)	0.003*
Rural	25.2 (31.3-45.5)	38.1 (31.3-45.5)	0.004*	48.4 (41.8-55.1)	31.0 (25.7-36.9)	0.001*	39.7 (32.7-47.2)	35.3 (30.4-40.6)	0.289

*p-value <0.05 (tested between 2004 and 2014 by Wald test)

The majority of the hypertensive cases were not aware of their condition in both 2004 and 2014 (Table 2). However, the awareness increased significantly from 2004 (19.4%) to 2014 (27.8%). A sub-group analysis by gender and location showed a similar improvement in the awareness of being hypertensive, with a tendency to larger improvements in rural than in urban areas, especially among rural females. Female were significantly more aware of their hypertension than males (Table 2), whereas there were no significant differences between urban and rural areas.

The proportion under treatment among those diagnosed with hypertension declined slightly, but not significantly, from 2004 (43.1%) to 2014 (40.1%), with an exception among rural females, who showed an increase from 38.0% to 45.9% (Table 2). Apart from rural females, no significant differences in treatment rates between urban-rural areas, or by gender, were detected between 2004 and 2014.

Among hypertensives, the proportion taking traditional medicines changed significantly, from 10.4% (0.80-13.3) in 2004 to 17.4% (14.3-21.0) in 2014 (not shown in tables).

Among the treated participants, the control rate did not improve from 2004 (48.4%) to 2014 (45.3%) (Table 2). In subgroup analyses, we found an increasing control rate from 2004 to 2014 among rural males, while the control rate decreased significantly among both urban and rural females (Table 2).

Among hypertensives who took traditional medicine, the control rate remained unchanged at 15.0% (10.9-20.4) in 2004 and 14.8% (10.7-20.1) in 2014 (not shown in tables).

From 2004 to 2014, the age-standardized mean systolic blood pressure increased 5.3 (SE) ± 0.97 mmHg, whereas diastolic blood pressure increased 4.7 ± 0.63 mmHg (Table 3). Similarly, subgroup analysis by location and gender revealed the same pattern of increase from 2004 to 2014 (Table 3).

**Table 3:** Age-standardized systolic and diastolic blood pressure among 25-74-year-old citizens in Yangon Region, Myanmar, in 2004 and 2014, by gender and location (Standardized to the 2014 Census Data of Yangon Region)

	Systolic Blood pressure			Diastolic Blood Pressure		
	2004 Mean(SE)	2014 Mean(SE)	*p*-value	2004 Mean(SE)	2014 Mean(SE)	*p*-value
Male	124.8 (0.88)	130.2 (0.83)	<0.001*	76.3 (0.60)	81.5 (0.63)	<0.001*
Female	121.2 (0.87)	126.2 (0.99)	<0.001*	76.1 (0.37)	80.3 (0.73)	<0.001*
Total	122.8 (0.82)	128.1 (0.53)	<0.001*	76.2 (0.35)	80.9 (0.53)	<0.001*
Urban						
Male	124.4 (1.27)	130.4 (0.95)	0.002*	77.2 (0.86)	82.1 (0.92)	0.001*
Female	120.7 (1.29)	126.3 (1.49)	0.014*	76.2 (0.48)	80.4 (1.10)	0.003*
Total	122.2 (1.22)	127.9 (0.79)	0.001*	76.6 (0.48)	81.2 (0.78)	<0.001*
Rural						
Male	125.1 (1.25)	129.3 (0.26)	0.014*	74.4 (0.75)	80.0 (0.35)	<0.001*
Female	121.8 (0.52)	125.1 (0.84)	0.008*	75.9 (0.54)	79.9 (0.36)	<0.001*
Total	123.7 (0.83)	127.4 (0.42)	0.001*	75.4 (0.39)	80.0 (0.21)	<0.001*

*p-value <0.05, by Wald test- for mean differences between 2004 and 2014

In multivariate analyses of 2004 data, age, alcohol consumption, low physical activity, overweight, diabetes and hypercholesterolemia were all significantly associated with hypertension, thereby indicating direct associations between these variables and hypertension (Table 4, Model 4). Similar associations were found in 2014, but not for low physical activity and hypercholesterolemia (Table 5, Model 4). Underweight was negatively associated with hypertension in both 2004 and 2014 (Tables 4 and 5, Model 4). Unexpectedly, smoking was not associated with hypertension in the fully adjusted model (Tables 4 and 5, Model 4), or when adjusted for socio-demographic factors (Tables 4 and 5, Model 1). No interaction of variables related to hypertension was found.

**Table 4:** Association between hypertension and selected socio-demographic-, behavioural- and metabolic risk factors among 25-74-year-old citizens in Yangon Region, Myanmar, in 2004

		Model 1		Model 2		Model 3		Model 4	
		pOR (95% CI)	*p*-value	pOR (95% CI)	*p*-value	pOR (95% CI)	*p*-value	pOR (95% CI)	*p*-value
Socio-demographic:									
Age		1.06 (1.06-1.07)	<0.001*	1.07 (1.06-1.08)	<0.001*	1.06 (1.05-1.08)	<0.001*	1.07 (1.05-1.08)	<0.001*
Location	Urban	(Ref)		(Ref)		(Ref)		(Ref)	
	Rural	0.85 (0.64-1.14)	0.262	0.89 (0.66-1.19)	0.394	1.05 (0.79-1.39)	0.726	1.08 (0.82-1.42)	0.546
Gender	Male	(Ref)		(Ref)		(Ref)		(Ref)	
	Female	1.15 (1.01-1.31)	0.032*	1.20 (1.00-1.43)	0.047*	0.98 (0.84-1.14)	0.728	1.08 (0.89-1.32)	0.386
Education	No formal	(Ref)		(Ref)		(Ref)		(Ref)	
	1-5 years	1.34 (0.92-1.95)	0.116	1.38 (0.95-1.99)	0.085	1.10 (0.74-1.64)	0.622	1.13 (0.75-1.71)	0.525
	6-11 years	1.48 (0.98-2.24)	0.062	1.46 (0.98-2.16)	0.059	1.11 (0.70-1.77)	0.622	1.17 (0.74-1.86)	0.463
	> 11 years	1.42 (0.91-2.22)	0.113	1.36 (0.87-2.13)	0.163	1.00 (0.63-1.57)	0.987	1.03 (0.64-1.64)	0.903
Ethnicity	Bamar	(Ref)		(Ref)		(Ref)		(Ref)	
	Non-Bamar	1.01 (0.80-1.29)	0.901	0.97 (0.75-1.25)	0.783	0.91 (0.69-1.20)	0.495	0.91 (0.71-1.18)	0.450
Behavioural:									
Tobacco smoking	No	(Ref)		(Ref)				(Ref)	
	Yes	0.83 (0.69-1.01)	0.069	0.78 (0.65-0.94)	0.013*			0.97 (0.82-1.16)	0.738
Alcohol consumption	No	(Ref)		(Ref)				(Ref)	
	Yes	1.46 (1.12- 1.9)	0.009*	1.53 (1.18-1.99)	0.004*			1.64 (1.24-2.17)	0.002*
Low physical activity	No	(Ref)		(Ref)				(Ref)	
	Yes	1.82 (1.23-2.69)	0.005*	1.83 (1.23-2.69)	0.005*			1.91 (1.28-2.85)	0.004*
Metabolic:									
BMI	Normal	(Ref)				(Ref)		(Ref)	
	Underweight	0.42 (0.30-0.59)	<0.001*			0.44 (0.31-0.62)	<0.001*	0.43 (0.30-0.61)	<0.001*
	Overweight	2.02 (1.58-2.58)	<0.001*			1.88 (1.49-2.37)	<0.001*	1.89 (1.49-2.40)	<0.001*
Diabetes	No	(Ref)				(Ref)		(Ref)	
	Yes	1.94 (1.36-2.77)	<0.001*			1.61 (1.13-2.31)	0.013*	1.60 (1.10-2.32)	0.018*
Hypercholesterolemia	No	(Ref)				(Ref)		(Ref)	
	Yes	1.48 (1.20-1.82)	<0.001*			1.22 (0.99-1.48)	0.052	1.22 (1.00-1.49)	0.050*

Model 1 adjusted for age, location, gender, education and ethnicity; Model 2 adjustment –behavioural risk factors: smoking, alcohol consumption, low physical activity were added to Model 1; Model 3 adjustment- the metabolic risk factors: BMI, diabetes and hypercholesterolemia were added to Model 1; Model 4 included all variables in the table

*p-value ≤0.05 from multivariate logistic regression

**Table 5:** Association between hypertension and selected socio-demographic-, behavioural- and metabolic risk factors among 25-74-year-old citizens in Yangon region, Myanmar, in 2014

		Model 1		Model 2		Model 3		Model 4	
		pOR (95% CI)	*p*-value	pOR (95% CI)	*p*-value	pOR (95% CI)	*p*-value	pOR (95% CI)	*p*-value
Socio-demographic:									
Age		1.05 (1.04-1.07)	<0.001*	1.06 (1.04-1.07)	<0.001*	1.05 (1.03-1.07)	<0.001*	1.06 (1.04-1.07)	<0.001*
Location	Urban	(Ref)		(Ref)		(Ref)		(Ref)	
	Rural	0.97 (0.65-1.43)	0.862	0.93 (0.62-1.40)	0.710	1.18 (0.72-1.90)	0.470	1.14 (0.68-1.92)	0.587
Gender	Male	(Ref)		(Ref)		(Ref)		(Ref)	
	Female	1.09 (0.82-1.47)	0.505	1.27 (0.71-2.27)	0.382	0.80 (0.58-1.11)	0.163	1.03 (0.58-1.83)	0.900
Education	No formal	(Ref)		(Ref)		(Ref)		(Ref)	
	1-5 years	0.63 (0.30-1.30)	0.184	0.62 (0.31-1.24)	0.154	0.70 (0.31-1.60)	0.364	0.70 (0.32-1.56)	0.344
	6-11 years	0.74 (0.36-1.55)	0.389	0.69 (0.34-1.41)	0.278	0.84 (0.39-1.78)	0.619	0.83 (0.40-1.75)	0.590
	> 11 years	0.71 (0.32-1.60)	0.370	0.65 (0.31-1.37)	0.226	0.69 (0.29-1.64)	0.361	0.67 (0.28-1.59)	0.328
Ethnicity	Bamar	(Ref)		(Ref)		(Ref)		(Ref)	
	Non-Bamar	0.76 (0.58-0.99)	0.046*	0.74 (0.56-0.99)	0.045*	0.79 (0.60-1.04)	0.091	0.79 (0.61-1.02)	0.070
Behavioural:									
Tobacco smoking	No	(Ref)		(Ref)				(Ref)	
	Yes	0.72 (0.46-1.14)	0.146	0.64 (0.43-0.96)	0.035*			0.83 (0.67-1.04)	0.101
Alcohol consumption	No	(Ref)		(Ref)				(Ref)	
	Yes	1.84 (1.04-3.26)	0.039*	2.04 (1.23-3.38)	0.011*			2.01 (1.09-4.03)	0.030*
Low physical activity	No	(Ref)		(Ref)				(Ref)	
	Yes	0.96 (0.65-1.40)	0.833	0.96 (0.68-1.38)	0.826			0.94 (0.66-1.37)	0.751
Metabolic:									
BMI	Normal	(Ref)				(Ref)		(Ref)	
	Underweight	0.40 (0.22-0.71)	0.006*			0.41 (0.22-0.78)	0.011*	0.41 (0.21-0.80)	0.014*
	Overweight	3.09 (2.43-3.91)	<0.001*			2.95 (2.34-3.72)	<0.001*	2.93 (2.28-3.78)	<0.001*
Diabetes	No	(Ref)				(Ref)		(Ref)	
	Yes	1.83 (1.39-2.41)	0.001*			1.47 (1.04-2.07)	0.034*	1.49 (1.05-2.12)	0.029*
Hypercholesterolemia	No	(Ref)				(Ref)		(Ref)	
	Yes	1.27 (0.80-2.00)	0.277			1.15 (0.74-1.78)	0.496	1.17 (0.78-1.76)	0.410

Model 1 adjusted for age, location, gender, education and ethnicity; Model 2 adjustment –behavioural risk factors: smoking, alcohol consumption, low physical activity were

added to Model 1; Model 3 adjustment- the metabolic risk factors: BMI, diabetes and hypercholesterolemia were added to Model 1; Model 4 included all variables in the table

*p-value ≤0.05 from multivariate logistic regression

In the combined data set (2004 and 2014) (Table 6), the study year was significantly associated with hypertension when adjusted for age and gender (Model 1: prevelance odd ratio pOR = 1.49, 95% CI: 1.21-1.82), adjusted for all socio-demographic factors (age, gender, location, education and ethnicity) (Model 2: pOR = 1.49, 1.22-1.82), and when adjusted for socio-demographic- and behavioural factors (smoking, alcohol consumption, low physical activity) (Model 3: pOR = 1.44, 1.17-1.77). However, when the association between study-year and hypertension was adjusted for socio-demographic- and metabolic risk factors (BMI, diabetes and hypercholesterolemia), the point estimate decreased and was no longer statistically significant (Model 4: pOR = 1.28, 0.92-1.78). In the fully adjusted model (Model 5) with socio-demographic-, behavioural- and metabolic risk factors included, the association between year of study and hypertension contributing to the association was not significant (pOR = 1.22, 0.89-1.68).

**Table 6:** Association between hypertension and year of study (2004/2014), socio-demographic-, behavioural- and metabolic risk factors in 2004 and 2014 combined

		Model 1		Model 2		Model 3		Model 4		Model 5	
		pOR (95% CI)	*p*-value	pOR (95% CI)	*p*-value	pOR (95% CI)	p-value	pOR (95% CI)	*p*-value	pOR (95% CI)	*p*-value
Socio-demographic:											
Year	2004	(Ref)		(Ref)		(Ref)		(Ref)		(Ref)	
	2014	1.49 (1.21-1.82)	0.001*	1.49 (1.22-1.82)	<0.001*	1.44 (1.17-1.77)	0.001*	1.28 (0.92-1.78)	0.129	1.22 (0.89-1.68)	0.207
Age		1.06 (1.05-1.06)	<0.001*	1.05 (1.04-1.07)	<0.001*	1.06 (1.05-1.07)	<0.001*	1.05 (1.04-1.07)	<0.001*	1.06 (1.05-1.07)	<0.001*
Location	Urban			(Ref)		(Ref)		(Ref)		(Ref)	
	Rural			0.94 (0.72-1.23)	0.629	0.92 (0.69-1.22)	0.535	1.15 (0.83-1.58)	0.385	1.12 (0.79-1.59)	0.496
Gender	Male	(Ref)		(Ref)		(Ref)		(Ref)		(Ref)	
	Female	1.09 (0.88-1.35)	0.406	1.10 (0.89-1.36)	0.376	1.24 (0.85-1.81)	0.254	0.85 (0.67-1.08)	0.171	1.05 (0.72-1.53)	0.78
Education	No formal			(Ref)		(Ref)		(Ref)		(Ref)	
	1-5 years			0.79 (0.49-1.26)	0.304	0.78 (0.49-1.25)	0.293	0.78 (0.46-1.33)	0.35	0.78 (0.46-1.32)	0.346
	6-11 years			0.89 (0.55-1.44)	0.623	0.86 (0.53-1.38)	0.511	0.88 (0.54-1.44)	0.597	0.88 (0.54-1.43)	0.584
	> 11 years			0.65 (0.50-1.44)	0.529	0.80 (0.47-1.36)	0.388	0.75 (0.42-1.32)	0.303	0.73 (0.41-1.29)	0.268
Ethnicity	Bamar			(Ref)		(Ref)		(Ref)		(Ref)	
	Non-Bamar			0.81 (0.68-0.98)	0.030*	0.80 (0.66-0.96)	0.021*	0.81 (0.68-0.98)	0.029*	0.81 (0.68-0.96)	0.017*
Behavioural:											
Tobacco smoking	No					(Ref)				(Ref)	
	Yes					0.67 (0.52-0.88)	0.006*			0.87 (0.74-1.01)	0.061
Alcohol consumption	No					(Ref)				(Ref)	
	Yes					1.92 (1.33-2.76)	0.001*			2.00 (1.26-3.17)	0.005*
Low physical activity	No					(Ref)				(Ref)	
	Yes					1.10 (0.81-1.50)	0.517			1.01 (0.81-1.49)	0.532
Metabolic:											
BMI	Normal							(Ref)		(Ref)	
	Underweight							0.43 (0.29-0.64)	<0.001*	0.42 (0.28-0.64)	<0.001*
	Overweight							2.62 (2.17-3.17)	<0.001*	2.62 (2.14-3.20)	<0.001*
Diabetes	No							(Ref)		(Ref)	
	Yes							1.49 (1.16-1.91)	0.003*	1.50 (1.16-1.93)	0.003*
Hypercholesterolemia	No							(Ref)		(Ref)	
	Yes							1.16 (0.86-1.56)	0.317	1.17 (0.88-1.54)	0.264

Model 1 adjusted for age and gender; Model 2 adjusted for age, gender, location, education and ethnicity; Model 3 adjustment- the behavioural risk factors: smoking, alcohol consumption and low physical activity were added to Model 2; Model 4 adjustment- the metabolic risk factors: BMI, diabetes and hypercholesterolemia were added to Model 2; Model 5 included all variables in the table

To further explore which of the metabolic risk factors influenced the association between study year and hypertension, we made six new models of different combinations of one or two metabolic risk factors in a model, together with the selected socio-demographic factors (Additional file 1: Table S1). The only combination that made the association between study year and hypertension non-significant was the combination with socio-demographic factors and BMI and hypercholesterolemia (Additional file 1: Table S1, Model 4e: pOR = 1.31 (0.96-1.78). No interaction between gender and smoking, and gender and location was detected.

## Discussion

The prevalence of hypertension, as well as the mean blood pressure levels, rose from 2004 to 2014 in both the urban and rural areas of the Yangon Region. The awareness, treatment and control rate of hypertension was still low in 2014, although the awareness of hypertension increased in urban and rural areas among both males and females. However, the treatment rate only improved among rural females. The control rate improved among urban and rural males, and decreased among both urban and rural females. Factors associated with hypertension in both 2004 and 2014 were age, alcohol consumption, overweight and diabetes. In 2004, low physical activity and hypercholesterolemia were also associated with hypertension.

An adjustment for socio-demographic- and behavioural factors did not change the association between study year and hypertension, while the combination of socio-demographic factors, BMI and hypercholesterolemia made the association non-significant.

### Strengths and limitations

Bias related to selection and information retrieval is relevant for both prevalence estimates and association measures. The studies had high response rates, hence minimizing the risk of selection bias. The exclusion of pregnant women and hospitalized people from the study may have contributed to more precise population estimates since we excluded individuals with a health situation affecting measurements such as blood pressure. We also excluded military personnel, Buddhist monks and nuns, who may have lifestyles that differ from the general citizens because of physical training among soldiers and religious customs in the monasteries, possibly introducing selection bias. However, it is difficult to conclude in which direction the results may have been biased. Difference in some selected townships of 2004 and 2014 study may have introduced a selection bias, however we do not expect systematic differences between townships due to representativeness from random sampling. The interviewing strategy in both studies might have also introduced information bias. However, we followed standardized WHO STEPS methodology in training and the use of instruments, thereby minimizing the potential for such bias. For anthropometric measurements, we used WHO-recommended OMRON devices, but the models were not the same in the two studies. Nonetheless, the devices were standardized in each study in order to minimize the potential information bias. Recall bias may have particularly occurred in questions about self-reported behavioural risk factors. Both studies had a single visit for BP readings, which may have potentially over- or underestimated the prevalence and control estimates [20]. However, such bias is probably randomly distributed in both 2004 and 2014. We did not include instruments in the studies for measuring the daily intake of salt and stress level, which are known risk factors for hypertension, and could have contributed to explaining the increase in hypertension from 2004 to 2014.

The current finding of a high prevalence of hypertension, increasing from 2004 to 2014, may be still on the rise; consequestnly, the poor levels of awareness, treatment and control pose a serious public health problem in Myanmar. Similar patterns have been reported in developing countries despite the fact that global estimates decreased between 1980 and 2008 for both genders [21]. Studies have confirmed that the prevalence of hypertension in Southeast Asian countries are on the rise [9], while a recent report indicates that the prevalence of hypertension in the entire Southeast Asia region has not decreased [3].

The increased age-standardized mean systolic- and diastolic blood pressure in the present study are in line with the regional southeast Asia figures of increases of 0-9 mmHg in males and higher than 1-3 mmHg in females per decade [22], although the global systolic blood pressure has decreased slightly [22].

Since 2011, the political situation in Myanmar has changed. The rural-urban migration has accelerated, and dietary patterns have become unhealthier, thus leading to increased body weights [23], which are highly associated with high blood pressure. Population growth and ageing [3], as well as a modern stressful environment may also the enhance the prevalence of hypertension. Moreover, the people of Myanmar enjoy salty food. For instance, Nga-pi (salty fish paste) is essential in Burmese dishes. The increasing trend in the prevalence of hypertension is possibly caused by a combination of urbanization, high salt intake, an unhealthy diet the included unhealthy fat, weight gain, hardship and the stress of a modern lifestyle.

Similar to findings from a systematic meta-analysis [24], there was a significant increase in the awareness of hypertension over the decade from 2004 to 2014, but the awareness remained far from the rule of halves [24]. About two-thirds of hypertensives were not aware, and this poor awareness is possibly caused by the poor education and poor adherence to primary health care. The other possible explanation is that the people of Myanmar might hesitate to go a health facility to see doctors and health worker until the symptoms have become serious. By nature, hypertension is asymptomatic in the early stages. A higher awareness proportion was observed among females, who may have more concerns about health in general than males, which is shown in studies from other countries [24, 25].

There was no change in treatment rate in the present study, although the rate is low, roughly below half of the diagnosed subjects. This could possibly be explained by the cost of antihypertensive medication, which is a burden to people living in developing countries [26]. The majority of the study population had a low household income, which could also contribute to a poor treatment rate. The majority of countries in Southeast Asia revealed a poor coverage of anti-hypertensive treatment [9].

Guidelines for anti-hypertensive treatment have not been well developed in Myanmar, although anti-hypertensive drugs are on the list of essential medicines [27]. Both in the rural and urban areas in Myanmar, people believe in traditional medicines for hypertension, as some studies have proven the effectiveness of traditional anti-hypertensive medicine [28, 29]. The National NCD control programme should consider effective anti-hypertensive treatment strategies, and drug policies and a procurement system to optimize the effect of the treatment. The low treatment and control rates highlight the importance of a hypertension control programme. Accessibility, affordability and an adherence of anti-hypertension treatment in Myanmar should be reviewed, and an effective control programme should be implemented.

Similar to a national study in Myanmar from 2009, we found that hypertension was associated with both behavioural- and metabolic factors [30]. In accordance with the general literature, we report an association between hypertension and alcohol consumption, low physical activity, overweight, diabetes and hypercholesterolemia in 2004 or 2014. Measures to improve these factors could be considered in a national programme to help reduce the prevalence of hypertension, and thus, the burden of the CVDs in Myanmar, in addition to investigating in more detail the salt intake and food habits of the people of Myanmar. Special attention should be paid towards a high BMI and hypercholesterolemia, as the two factors may have played in the important role for the increase in hypertension from 2004 to 2014 in the Yangon region, which is one of the most developed parts of Myanmar.

One should be careful in generalizing the results from this study to the entire country of Myanmar, as lifestyle and behavioural risk factors of the Yangon region, including Yangon City, which is the largest city in Myanmar, may differ from other parts of Myanmar. Even so, it is likely that changes in the prevalence of hypertension, awareness, treatment and control have changed in the same direction as in Yangon region during last decade. National studies are therefore needed to confirm this.

## Conclusion

The prevalence of hypertension, mean systolic and diastolic blood pressure is high, and has increased significantly in the Yangon region of Myanmar between 2004 and 2014. A high BMI and hypercholesterolemia may have contributed to the increased prevalence. The level of awareness, treatment and control must be improved in order to reduce future premature deaths and disability. An effective national hypertension control program is thus urgently needed.

## Additional file

Additional file 1:Table S1. Associations between study-year and hypertension, adjusted for sociodemographic factors and different combinations of metabolic risk factors, among 25-74-year-old citizens in Yangon Region, Myanmar, 2004 and 2014 combined. (XLSX 12 kb)

## Abbreviations

BMI: Body mass index
CVDs: Cardiovascular diseases
DALY: Disability-adjusted Life Year
DBP: Diastolic blood pressure
MET: Metabolic equivalent of tasks
NCD: Non-communicable diseases
POR: Prevalence odd ratio
SBP: Systolic blood pressure
SDGs: Sustainable Development Goals
STEPS: STEPwise approach to non-communicable disease risk factor surveillance

## References

[1] GBD 2015 Risk Factors Collaborators (2016). Global, regional, and national comparative risk assessment of 79 behavioural, environmental and occupational, and metabolic risks or clusters of risks, 1990-2015: a systematic analysis for the global burden of disease study 2015. Lancet.

[2] Lim SS, Vos T, Flaxman AD (2013). A comparative risk assessment of burden of disease and injury attributable to 67 risk factors and risk factor clusters in 21 regions, 1990–2010: a systematic analysis for the global burden of disease study 2010. Lancet.

[3] NCD Risk Factors Collaboration (NCD-RisC) (2016). Worldwide trends in blood pressure from 1975 to 2015: a pooled analysis of 1479 population-based measurement studies with 19.1 million participants. Lancet.

[4] GBD 2015 Mortality and Causes of Death Collaborators. Global, regional, and national life expectancy, all-cause mortality, and cause-specific mortality for 249 causes of death, 1980-2015: a systematic analysis for the global burden of disease study 2015. Lancet 2016; 388 (10053): 1459-1544. https://www.ncbi.nlm.nih.gov/pubmed/?term=Global%2C+regional%2C+and+national+life+expectancy%2C+all-cause+mortality%2C+and+cause-specific+mortality+for+249+causes+of+death%2C+1980-2015%3A+a+systematic+analysis+for+the+Global+Burden+of+Disease+Study+2015.+Lancet+2016%3B+388(10053)%3A+1459-544. Accessed 15 Jan 2017. 10.1016/S0140-6736(16)31012-1 PMC5388903 27733281

[5] Olsen MH, Angell SY, Asma S (2016). A call to action and a lifecourse strategy to address the global burden of raised blood pressure on current and future generations: the lancet commission on hypertension. Lancet.

[6] World Health Organization. The 66th world health assembly: second report of committee A66/65. Geneva. 2013:2013. http://apps.who.int/gb/ebwha/pdf_files/WHA66-REC1/WHA66_2013_REC1_complete.pdf Accessed 15 Jan 2017

[7] Mendis Shanthi. Global status report on noncommunicable diseases 2014: World Health Organization; 2014. http://apps.who.int/iris/bitstream/10665/148114/1/9789241564854_eng.pdf Accessed 15 Jan 2017. 10.1161/STROKEAHA.115.008097 25873596

[8] World Health Organization. Global health risks: mortality and burden of disease attributable to selected major risks: world health Organization; 2009. http://www.who.int/healthinfo/global_burden_disease/GlobalHealthRisks_report_full.pdf Accessed 15 Jan 2017.

[9] Krishnani A, Gargii R, Kahandaliyanageiii A (2013). Hypertension in the South-East Asia region: an overview. Regional Health Forum.

[10] WHO-SEAROffice. Non-communicable disease risk factor survey Myanmar 2009. 2011. http://www.who.int/chp/steps/2009_STEPS_Survey_Myanmar.pdf Accessed 15 Jan 2017.

[11] Zaw KK, Latt TS, Phyu Phyu A, Thein Gi T, Tin Khine M (2011). Prevalence of hypertension and its associated factors in the adult population in Yangon division, Myanmar Asia. Pac J Public Health.

[12] Htet AS, Bjertness MB, Sherpa LY (2016). Urban-rural differences in the prevalence of non-communicable diseases risk factors among 25-74 years old citizens in Yangon Region, Myanmar: a cross sectional study. BMC Public Health.

[13] World Health Organization. WHO STEPS surveillance manual: The WHO STEPwise approach to chronic disease risk factor surveillance. 2008. http://www.who.int/chp/steps/manual/en/ Accessed 15 Jan 2017.

[14] Topouchian J, Agnoletti D, Blacher J (2014). Validation of four devices: Omron M6 comfort, Omron HEM-7420, Withings BP-800, and Polygreen KP-7670 for home blood pressure measurement according to the European Society of Hypertension International Protocol. Vasc Health Risk Manag.

[15] The ward or village tract administration Law 2012. The Ministry of Home Affair, the Republic of the Union of Myanmar; 2012. http://www.gad.gov.mm/sites/default/files/laws-rules/2013/12/8._village_act.pdf Accessed 15 Jan 2017.

[16] World Health Organization. Definition and diagnosis of diabetes mellitus and intermediate hyperglycemia : Report of a WHO/IDF consultation.; 2006. http://apps.who.int/iris/bitstream/10665/43588/1/9241594934_eng.pdf Accessed 15 Jan 2017.

[17] Third Report of the National Cholesterol Education Program (NCEP) Expert Panel on Detection, Evaluation, and Treatment of High Blood Cholesterol in Adults (Adult Treatment Panel III) final report, 2002. http://circ.ahajournals.org/content/106/25/3143.long Accessed 15 Jan 2017. 12485966

[18] Central Statistical Organization. Statistical Yearbook 2004. Yangon, Myanmar: Central Statistical Organization, 2005.

[19] Department of Population, Ministry of Immigration and Population. The 2014 Myanmar Population and Housing Census: Yangon Region Census Report, 2015. http://www.dop.gov.mm/. Accessed 15 Jan 2017.

[20] Birkett NJ (1997). The effect of alternative criteria for hypertension on estimates of prevalence and control. J Hypertens.

[21] Ibrahim MM, Damasceno A (2012). Hypertension in developing countries. Lancet.

[22] Danaei G, Finucane MM, Lin JK (2011). National, regional, and global trends in systolic blood pressure since 1980: systematic analysis of health examination surveys and epidemiological studies with 786 country-years and 5.4 million participants. Lancet.

[23] Ebrahim S, Kinra S, Bowen L (2010). The effect of rural-to-urban migration on obesity and diabetes in India: a cross-sectional study. PLoS Med.

[24] Marques-Vidal P, Tuomilehto J (1997). Hypertension awareness, treatment and control in the community: is the 'rule of halves' still valid?. J Hum Hypertens.

[25] Pereira M, Lunet N, Azevedo A, Barros H (2009). Differences in prevalence, awareness, treatment and control of hypertension between developing and developed countries. J Hypertens.

[26] Strasser T (1992). Relative costs of antihypertensive drug treatment. J Hum Hypertens.

[27] Ministry of Health, Myanmar. Essential and complementary medicines and vaccines for Myanmar. Myanmar Essential Medicines Project; 2010. http://www.moh.gov.mm/file/NLEM.pdf Accessed 15 Jan 2017.

[28] Khine Khine Lwin MAT, Khin Thet Wai, Nu Nu Win, Tun Myint Aye, San Yu Aung, San San Myint, Phyu Phyu Win. The use of herbal medicines and traditional medicine formulations (TMFs) for hypertension at traditional medicine hospital, Yangon. The Myanmar Health Sciences Research Journa*l* 2014; 23, Number 1: 41-47. http://dmrlm.gov.mm/publication/MHSRJ/Current%20Issue/26%20_1%20whole%20book.pdf Accessed 15 Jan 2017.

[29] Khine Khine Lwin MAT, Kyaw T, Htay TT, Zaw KK, Myint KTY, Win NN, Maw WW (2014). Antihypertensive effect of Millingtonia hortensis Linn. f. Leaves (Aykayit) on stage 1 hypertensive patients. The Myanmar Health Sciences Research Journal.

[30] Bjertness MB (2016). Aung Soe Htet, Meyer HE, et al. prevalence and determinants of hypertension in Myanmar - a nationwide cross-sectional study. BMC Public Health.

